# A collision tumor of buccal squamous cell carcinoma and diffuse large B-cell lymphoma: a case report and literature review

**DOI:** 10.3389/fonc.2026.1912416

**Published:** 2026-09-03

**Authors:** Xiao Pang, Ying Long, Hui Wang, Zhang Zhang

**Affiliations:** 1 Hunan provincial clinical research center for Oncoplastic Surgery, The Affiliated Cancer Hospital of Xiangya School of Medicine, Hunan Cancer Hospital, Central South University, Changsha, Hunan, China; 2 Department of Radiation Oncology, The Affiliated Cancer Hospital of Xiangya School of Medicine, Hunan Cancer Hospital, Central South University, Changsha, Hunan, China; 3 Department of Pathology, The Affiliated Cancer Hospital of Xiangya School of Medicine, Hunan Cancer Hospital, Central South University, Changsha, Hunan, China

**Keywords:** buccal squamous cell carcinoma, case report, collision tumor, diffuse large B-cell lymphoma, MYC rearrangement, oral squamous cell carcinoma, PET-CT, polatuzumab vedotin

## Abstract

**Background:**

Collision tumors are rare entities in which two histologically distinct malignancies coexist in close proximity without histological admixture or transition. The coexistence of buccal squamous cell carcinoma (SCC) and diffuse large B-cell lymphoma (DLBCL) in the oral region is exceptionally uncommon. Because the clinical and radiological findings may overlap, such cases can pose substantial diagnostic and therapeutic challenges.

**Case presentation:**

We report a case of a 66-year-old man with a long history of smoking and betel quid chewing who presented with a rapidly enlarging mass in the left buccal region. Biopsy at a local hospital showed moderately to well-differentiated SCC. However, subsequent imaging revealed widespread, discontinuously distributed hypermetabolic lesions involving the thyroid gland, cervical and mediastinal lymph nodes, and terminal ileum, a pattern inconsistent with the usual lymphatic spread of buccal SCC. Repeated minimally invasive biopsies of cervical and thyroid lesions failed to establish a definitive diagnosis of lymphoma, partly because of prominent reactive lymphoid infiltration in the setting of Hashimoto’s thyroiditis. After multidisciplinary discussion, radical resection of the locally advanced buccal tumor was prioritized because of the risk of local progression and loss of resectability. Final histopathological examination of the surgical specimen demonstrated a collision tumor composed of independent SCC and DLBCL components. Immunohistochemistry and fluorescence *in situ* hybridization identified the lymphoma as non-germinal center B-cell-like DLBCL with isolated MYC rearrangement. The patient subsequently received Pola-R-CHP immunochemotherapy and achieved a favorable early response.

**Conclusion:**

This case highlights the need to consider a second primary malignancy when the distribution of lesions does not follow the expected metastatic pathway of head and neck SCC. It also illustrates the limitations of needle biopsy in lymphoid lesions arising in a background of chronic inflammation. Adequate tissue sampling, careful pathological review, PET-CT-based systemic assessment, and multidisciplinary decision-making are essential for accurate diagnosis and individualized treatment of complex collision tumors.

## Introduction

Multiple primary malignancies (MPMs) are defined as two or more independent primary malignant tumors arising synchronously or metachronously in one or more organs in the same patient (1). The concept was first put forward by Billroth in 1889 (2), and the currently widely adopted diagnostic criteria were formulated by Warren and Gates in 1932 (1): each tumor must show definite malignant histopathology (2); the tumors must arise at anatomically distinct sites; and (3) the possibility that one lesion represents metastasis from another must be excluded (3). According to the interval between tumor diagnoses, MPMs are generally classified as synchronous when the second primary tumor is detected within 6 months after the first primary cancer, and metachronous when the interval exceeds 6 months (4). Among patients with head and neck malignancies, MPMs are relatively common, with a reported incidence of approximately 2% to 6%; multiple squamous cell carcinomas of the respiratory or digestive tract are the most frequent pattern (5, 6). This phenomenon is largely explained by the field cancerization theory proposed by Slaughter et al. in 1953 (7). According to this theory, long-term exposure of the upper aerodigestive tract mucosa to shared carcinogens such as tobacco, alcohol, and betel nuts can induce widespread subclinical genetic alterations, creating a premalignant field that is susceptible to multicentric carcinogenesis and the development of multiple squamous cell carcinomas (SCCs).

By contrast, a collision tumor is an exceptionally rare entity in which two malignancies of distinct histogenetic origins meet within the same organ or tissue (8). It differs from a composite tumor, in which a single neoplasm undergoes divergent differentiation. A collision tumor represents the incidental spatial convergence of two independent clonal processes. The diagnostic hallmark is close apposition, or even interdigitation, of two tumors with different biological identities, without admixture of their components or a histologic transition zone (9, 10). The clinical behavior of a collision tumor is usually driven by its more aggressive component. For example, when a high-grade carcinoma coexists with a low-grade neoplasm, the high-grade component typically determines outcome and treatment (11, 12). Because the biological behavior of each component may differ substantially, and because one component often dominates the clinical presentation or biopsy findings, the second component may be missed, leading to major errors in treatment planning (8, 13). Here, we report a rare collision tumor arising in the left buccal region. The patient presented with moderately to well-differentiated squamous cell carcinoma of epithelial origin and diffuse large B-cell lymphoma (DLBCL) of mesenchymal origin. Based on clinical data, laboratory examinations and imaging findings, we clarify the characteristics of this case and mainly analyze key challenges in early diagnosis, such as imaging interference caused by inflammatory lesions and sampling limitations of minimally invasive biopsy. We also summarize clinical experience and discuss individualized diagnosis and treatment strategies, aiming to provide a reference for the clinical management of similar rare cases.

## Case presentation

**Figure 1:**
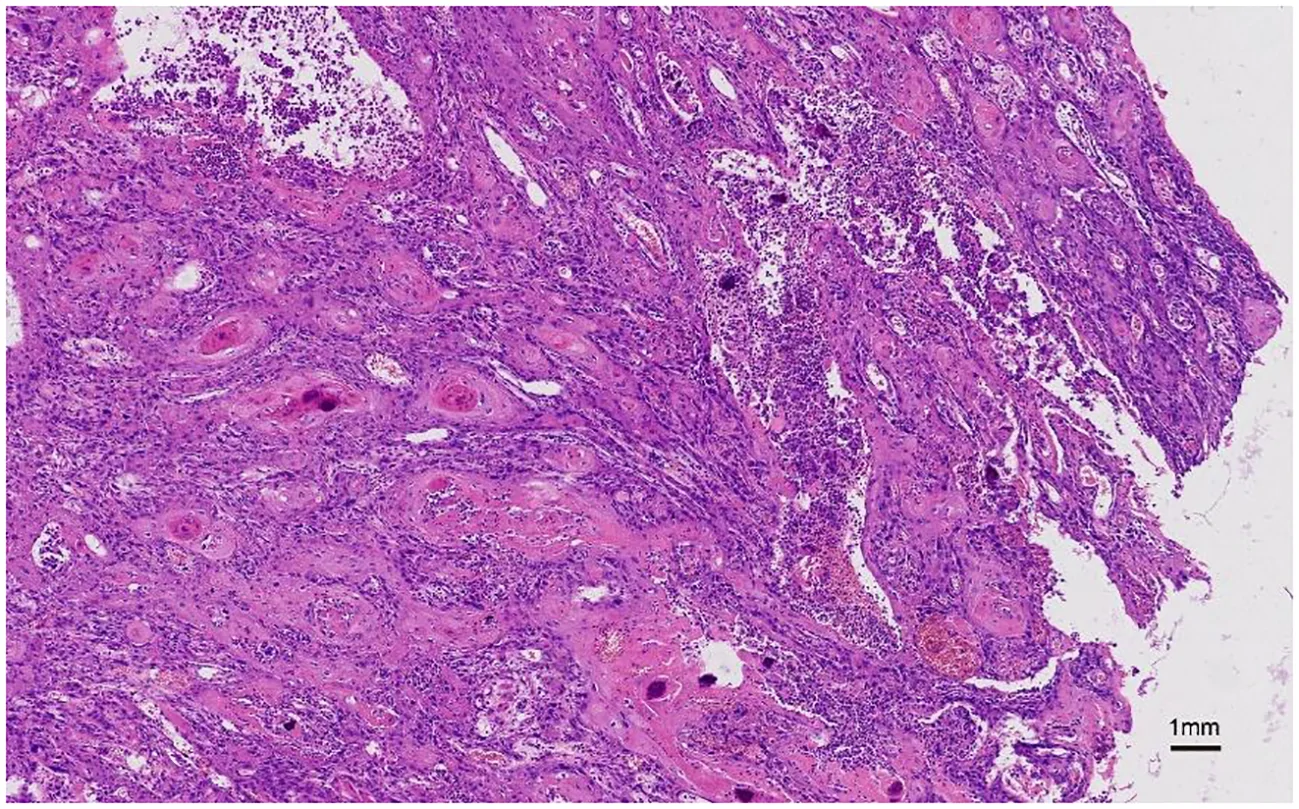
Representative images of biopsy lesions from local hospitals displayed under microscopic examination. A well-differentiated buccal squamous cell carcinoma with keratin pearl formation (H&E, 100×).

A 66-year-old man first noticed a soybean-sized mass in the left buccal region at the end of 2024. The lesion was initially asymptomatic. He took traditional Chinese medicine and anti-inflammatory drugs intermittently for about 10 months. The mass failed to resolve and underwent explosive enlargement, with mild local tenderness on palpation. On October 27, 2025, a biopsy of the left buccal lesion was performed at Xiangtan Central Hospital. Pathology consultation at our center on October 30, 2025, showed high differentiated squamous carcinoma of epithelial tissue (**Figure 1**). Because this was a superficial biopsy specimen, the invasive front was not included and the depth of invasion could not be reliably assessed before resection. The patient had long-term carcinogen exposure: a 50-year smoking history of about one pack daily, and a 4-year history of betel quid chewing with 10–12 pieces per day. These physical and chemical irritants are well-established major risk factors for oral squamous cell carcinoma (14) and may also promote lymphoproliferative disease by inducing a chronic inflammatory microenvironment (15–17).

**Figure 2:**
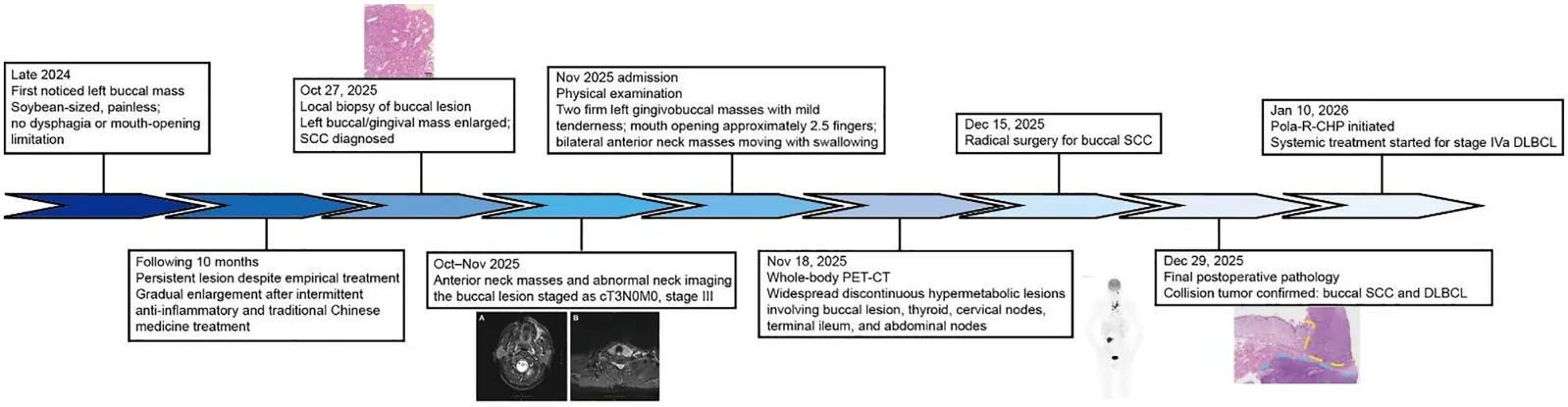
Timeline of symptom progression, diagnostic evaluation, final diagnosis, and treatment.

In November 2025, the patient was referred to our center for further evaluation and treatment. On admission, his general condition was fair, without obvious fever, night sweats or weight loss. Specialist examination revealed two firm, poorly demarcated masses involving the left gingiva and buccal mucosa, measuring 2.5 cm × 1.0 cm and 2.0 cm × 1.5 cm, respectively. The two lesions tended to coalesce and involved the left gingivobuccal sulcus, extending superiorly to the maxillary tooth positions 25 and 26 and inferiorly to the mandibular tooth position 36. Multiple enlarged cervical lymph nodes were palpable. Notably, one firm nodule measuring approximately 2.5 cm × 2.0 cm was present in each anterior cervical region and moved with swallowing, suggesting possible involvement of the thyroid or adjacent structures rather than the typical lateral neck nodal spread of SCC. Laboratory tests showed an elevated D-dimer level of 1.35 μg/mL FEU. Thyroid function tests suggested subclinical hypothyroidism, with an elevated thyroid-stimulating hormone level (TSH, 10.350 μIU/mL) and normal free triiodothyronine (FT3) and free thyroxine (FT4). Complete blood count, biochemical indices, and inflammatory markers showed no major abnormalities. Screening tests for syphilis, hepatitis B virus, hepatitis C virus, and human immunodeficiency virus were negative. Detailed laboratory results are shown in **Table 1**. The overall clinical course is summarized in **Figure 2**.

**Table 1:** Laboratory data.

Variable	Reference range, adults	On initial evaluation	After the surgery	After the chemotherapy
Red Blood Cell (×10¹²/L)	4.00-5.50	3.89	3.19	2.78
Hemoglobin (g/L)	120-160	115.00	95.00	80.00
White Blood Cell (×10^9^/L)	4.00-10.00	7.18	14.50	7.66
Lymphocyte Percentage (%)	20.00-40.00	19.10	5.50	8.70
Lymphocyte count (10^9^/L)	0.800-4.000	1.370	0.800	0.670
Platelet (×10^9^/L)	100-300	204	185	204
Alanine Aminotransferase (U/L)	9-50	16.10	30.40	10.80
Aspartate Aminotransferase (U/L)	15-40	20.30	15.80	21.50
Glucose(mmol/L)	3.9-6.1	5.33	5.10	5.40
Total Protein (g/L)	65.00-85.00	66.60	53.40	64.40
Albumin (g/L)	40.0-55.0	41.1	31.3	37.1
Serum Urea (mmol/L)	3.6-9.5	5.18	7.62	6.49
Serum Creatinine (μmol/L)	64-104	81.0	66.0	73.0
Serum Uric Acid (μmol/L)	208.3-428.4	370.8	158.0	276.2
Estimated Glomerular Filtration Rate (eGFR, mL/min)	–	88.4	97.5	93.5
Total cholesterol(mmol/L)	0.00-5.20	4.88	4.90	4.90
Triglyceride (mmol/L)	0.40-1.86	1.43	1.26	1.06
High density lipoprotein(mmol/L)	1.2-1.68	0.92	0.95	1.12
Low density lipoprotein(mmol/L)	<3.7	3.10	3.20	3.39
Lactate Dehydrogenase (U/L)	120-250	242.50	280.30	401.10
Serum Calcium (mmol/L)	2.20-2.65	2.29	2.10	2.00
D-Dimer (ug/mL FEU)	0.00-0.55	1.35	1.82	2.91
Fibrin Degradation Product (ug/mL)	0.00-5.00	–	–	8.54
Fibrinogen (g/L)	2.00-4.00	4.01	4.20	4.57
Thyroid Stimulating Hormone (μIU/mL)	0.56-5.91	10.350	9.870	9.500
Free Triiodothyronine (PG/ML)	2.30-4.80	3.520	3.480	3.400
Free Thyroxine (NG/DL)	0.62-1.24	0.635	0.640	0.645
C-Reactive Protein (mg/L)	0-6.0	2.34	42.34	11.26
Procalcitonin (ng/mL)	0-0.05	–	0.77	–
EB Virus DNA Quantitative Detection	<400 copies/mL	–	–	<400 copies/mL
Vascular Endothelial Growth Factor (VEGF) (pg/mL)	0.0-142.2	0.7	–	–
Squamous Cell Carcinoma Antigen (ng/mL)	0-2.7	2.1	3.5	2.8
Cytokeratin Fragment 21-1 (ng/mL)	0-3.5	2.8	4.2	3.5

**Figure 3:**
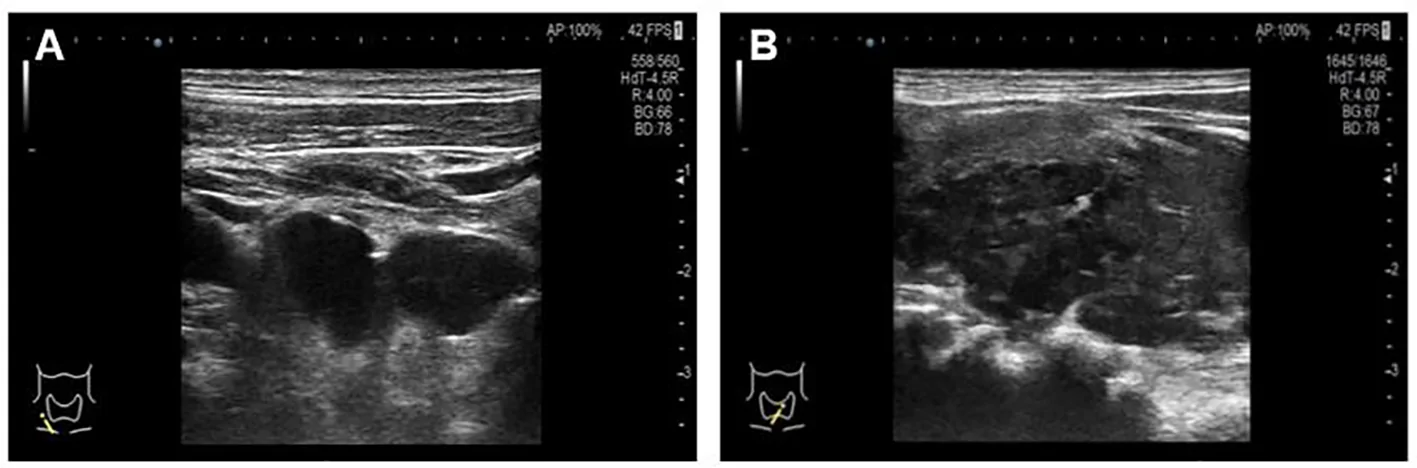
Neck ultrasound. **(A)** Transverse view of the left thyroid lobe: a 31 × 28 mm heterogeneous hypoechoic lesion with ill-defined borders. **(B)** Multiple enlarged lymph nodes are seen in level VI and pretracheal space.

Subsequent imaging findings, however, complicated the clinical picture. Neck color Doppler ultrasound revealed a heterogeneous hypoechoic area in the left thyroid lobe with an irregular shape and indistinct margins, classified as TI-RADS 5. Multiple enlarged lymph nodes were also seen in bilateral level VI regions, with heterogeneous internal echogenicity and posterior acoustic enhancement (**Figure 3**). Buccal SCC most commonly metastasizes to level I and level IIa cervical lymph nodes and rarely presents initially with level VI nodal or thyroid involvement (18). Moreover, because the primary buccal lesion was unilateral and laterally located, the presence of bilateral anterior cervical lymphadenopathy, including contralateral disease, was not readily explained by the usual nodal drainage pattern of buccal SCC. This distribution therefore raised the possibility of a second malignant process, such as a primary thyroid malignancy or an independent lymphoid disease.

**Figure 4:**
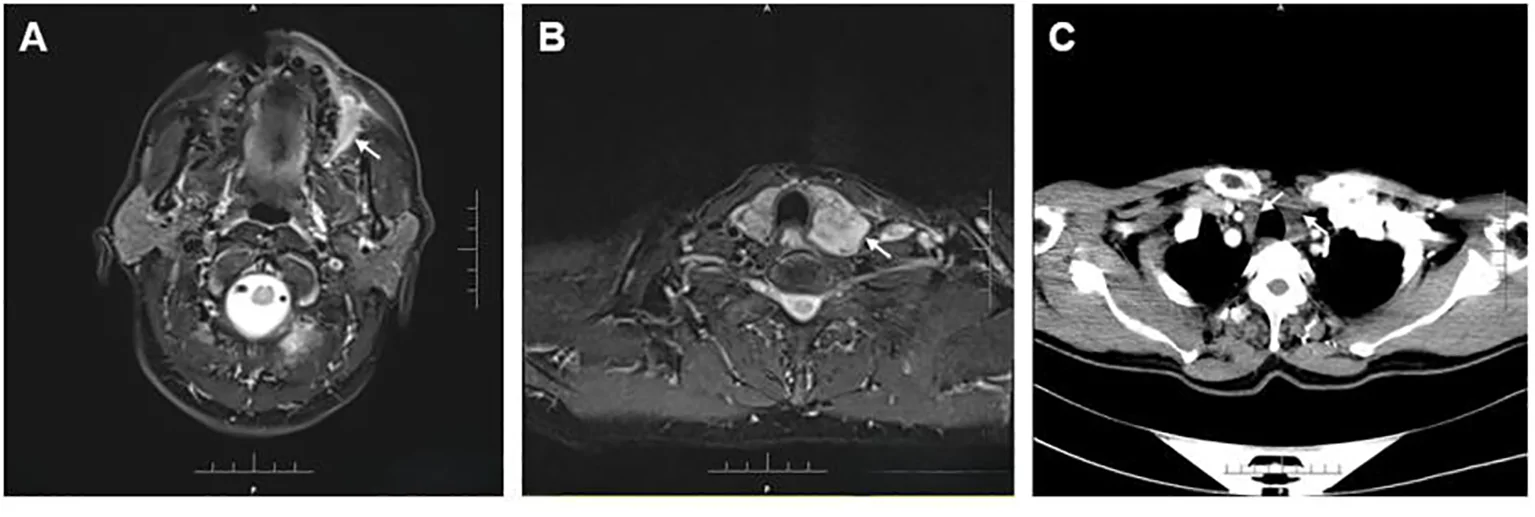
Neck MRI and CT images. **(A)** Primary lesion located in the left buccal region. **(B)** Enlarged thyroid gland with heterogeneous enhancement. **(C)** Multiple enlarged lymph nodes in level VI.

Subsequent thin-slice neck MRI and contrast-enhanced CT confirmed the extensive disease involvement. In addition to the primary left buccal lesion, the bilateral thyroid lobes were abnormally enlarged and contained multiple foci of abnormal density. Multiple enlarged lymph nodes were widely distributed in bilateral cervical levels IV, V, and VI and in the upper mediastinum (**Figure 4**). In view of this multicentric, cross-regional pattern, the clinical team considered the findings inconsistent with the usual metastatic route of advanced buccal SCC and highly suggestive of a second primary tumor.

**Figure 5:**
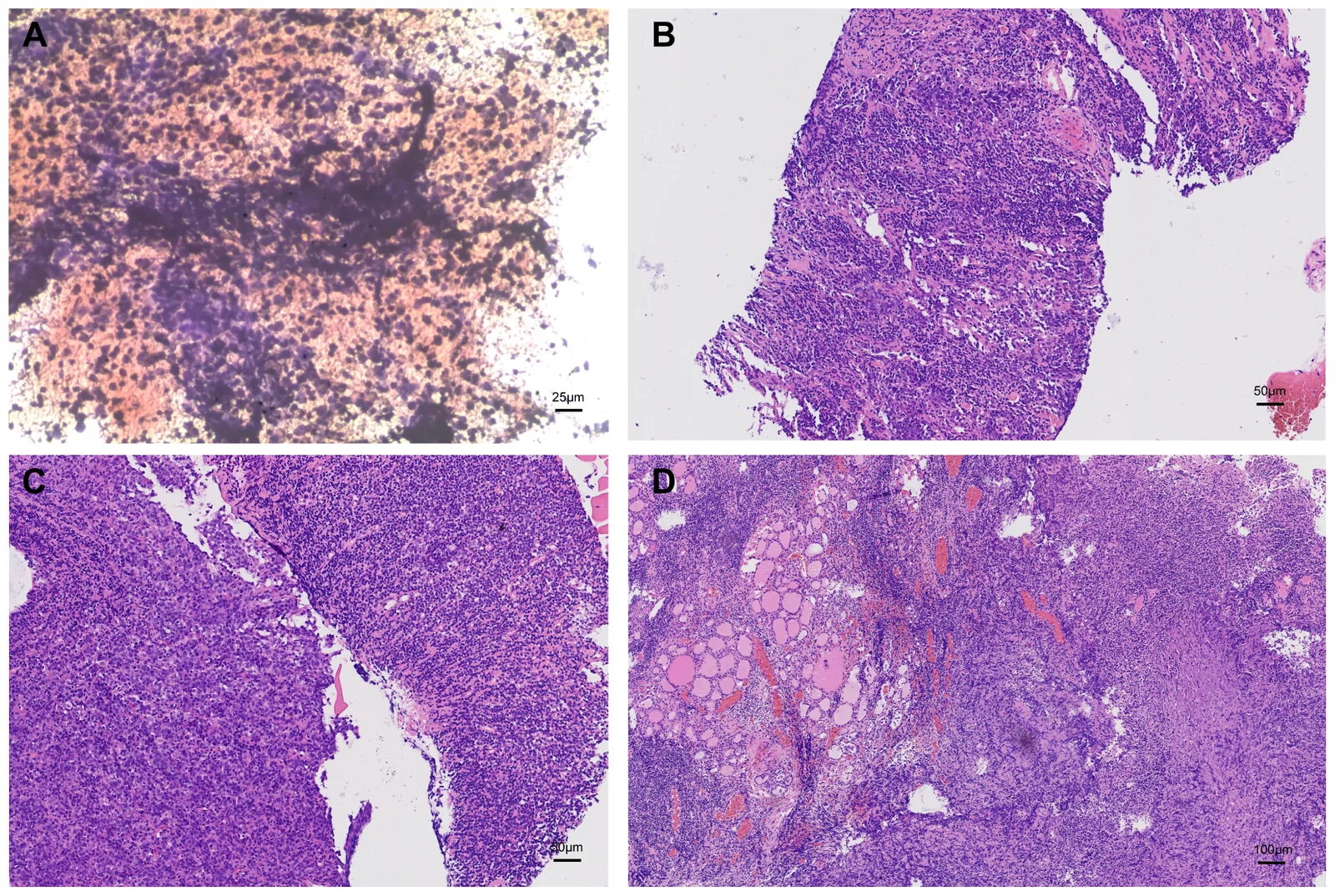
Pathological findings of neck lesions across different specimens. **(A)** Thyroid FNA cytology (Papanicolaou, 400×). No definitive thyroid parenchyma identified, with diffuse proliferation of heterogeneous lymphoid cells. **(B)** Thyroid core-needle biopsy histology (H&E, 200×). No normal thyroid tissue was seen, only diffuse lymphoid cell hyperplasia, the cell components were complex, and there had atypia. **(C)** Cervical lymph node biopsy histology (H&E, 200×). Diffuse lymphoid cell proliferation with effacement of normal nodal architecture. **(D)** Representative image of the left thyroid lesion removed by surgery under the microscope (H&E, 100×). Diffuse interspersed lymphoid proliferation in a background of atrophic thyroid tissue and fibroconnective hyperplasia, with mixed cellular components.

The clinical team reasoned that if concurrent systemic lymphoma could be confirmed, systemic medical therapy would be prioritized to avoid invasive maxillofacial resection and flap reconstruction surgery. To determine the pathological nature of cervical and thyroid lesions, the patient received repeated fine-needle aspiration (FNA) of cervical lymph nodes and core needle biopsy (CNB) of the thyroid. Although a limited biopsy sample was also obtained from a pretracheal cervical lymph node, the specimen was insufficient to establish lymphoma definitively. In retrospect, a targeted ultrasound- or PET-guided CNB, or complete excision biopsy of the most suspicious cervical lymph node would probably have provided more representative nodal architecture for lymphoma diagnosis than thyroid FNA/CNB in the setting of Hashimoto’s thyroiditis. Unfortunately, the results of thyroid and lymph node puncture examination only indicated a suspected lymphoproliferative lesion. The specimens showed sparse atypical cells and poor tissue architecture, and a definitive pathological diagnosis of hematolymphoid disease could not be made. FNA has limited accuracy for diagnosing lymphoma because of inadequate cellularity, sampling representativeness, and lack of tissue architecture. Similarly, even thyroid CNB may fail to establish lymphoma when sampling is insufficient or the disease is patchy, particularly when lymphoma cells are admixed with lymphocytes associated with Hashimoto’s thyroiditis. Considering the limitations of needle biopsy and remarkable thyroid lesions on imaging, the team performed surgical resection of the left thyroid lesion. This procedure provided more tissue than needle biopsy, but still did not establish a definitive diagnosis of lymphoma. Intraoperatively, a diffuse firm nodule measuring 3 cm × 3 cm was identified at the lower pole of the left thyroid lobe. After intradepartmental consultation for this difficult pathology case, the specimen showed diffuse, relatively uniform lymphoid-cell proliferation and infiltration in a background of Hashimoto’s thyroiditis. Some lymphoid cells showed some atypia and appeared to have a clonal growth pattern, with destruction of normal thyroid architecture. Nevertheless, the immunohistochemical profile was not typical, and B-cell receptor (BCR) gene rearrangement was negative. Therefore, the thyroid lesion was interpreted as Hashimoto’s thyroiditis with active and partially atypical lymphoid proliferation, rather than confirmed thyroid involvement by DLBCL. At this stage, there was still inadequate evidence to confirm a malignant hematolymphoid disorder, bringing further diagnosis and treatment to a standstill (**Figure 5**).

**Figure 6:**
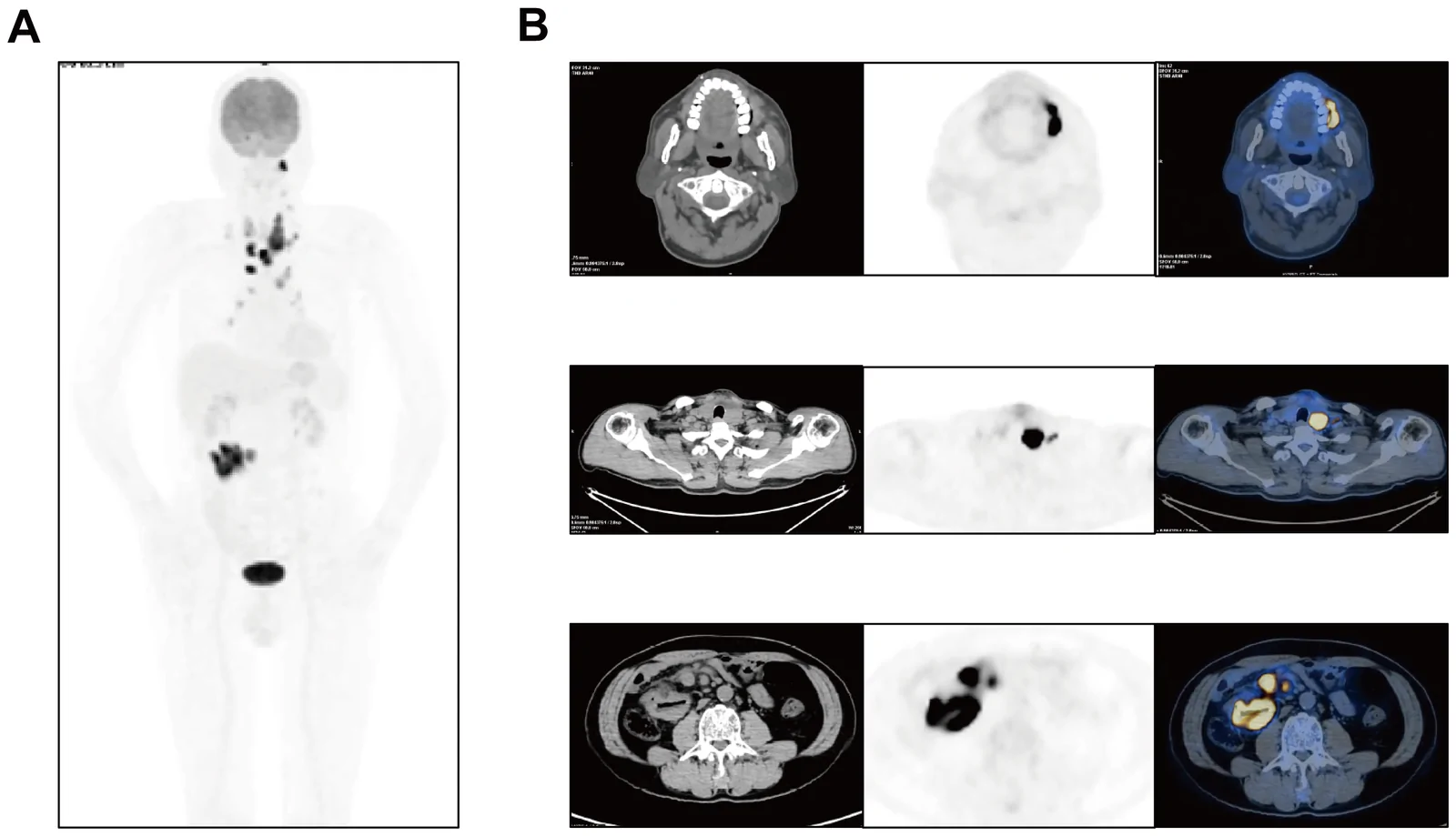
Whole-body ¹^8^;F-FDG PET-CT imaging. **(A)** Maximum-intensity projection (MIP) view. **(B)** Axial CT, PET, and fused PET/CT images at the levels of the buccal lesion, thyroid gland, and terminal ileum.

Because of this diagnostic dilemma, the patient underwent whole-body 18F-FDG PET-CT on November 18, 2025. The left buccal lesion showed intense hypermetabolism (SUVmax 28.4), consistent with SCC. In addition, the residual bilateral thyroid tissue showed diffuse abnormal uptake (SUVmax 23.4), accompanied by hypermetabolic lymph nodes in the bilateral neck, supraclavicular regions, and multiple mediastinal nodal stations (2R, 2L, 3A, and 6). Furthermore, intense uptake was identified in the thickened wall of the terminal ileum (SUVmax 27.1) and multiple lymph nodes in the right lower abdomen (SUVmax 31.3). The maximum-intensity projection (MIP) image clearly showed a widespread, discontinuous, “starry sky”-like pattern of metabolic activity (**Figure 6**), which was not compatible with the usual metastatic pattern of buccal SCC and suggested a second systemic malignancy.

This cross-regional, multi-organ pattern of extensive hypermetabolic lesions complicated clinical decision-making. PET-CT findings were highly suggestive of concurrent systemic lymphoma, particularly lesions within the terminal ileum and abdominal lymph nodes. Nevertheless, previous cervical lymph node FNA, thyroid CNB, and surgical specimen of thyroid lesion could not confirm lymphoma pathologically. Based on multidisciplinary assessment and available imaging findings, the buccal lesion was clinically staged as cT3N0M0, stage III, while the widespread non-contiguous lesions raised suspicion of a second primary malignancy. After multidisciplinary team (MDT) discussion, the buccal tumor was considered pathologically confirmed, clinically staged as T3, and locally aggressive, with an immediate risk of bleeding, airway compromise, or loss of resectability. The clinical team therefore decided to prioritize surgery to control the buccal primary tumor, with adjuvant radiotherapy to be reassessed postoperatively. After effective control of the buccal carcinoma, colonoscopy or surgical resection of the involved bowel could be considered to characterize the intestinal lesions. If lymphoma was confirmed, subsequent treatment would be guided by the lymphoma subtype.

**Figure 7:**
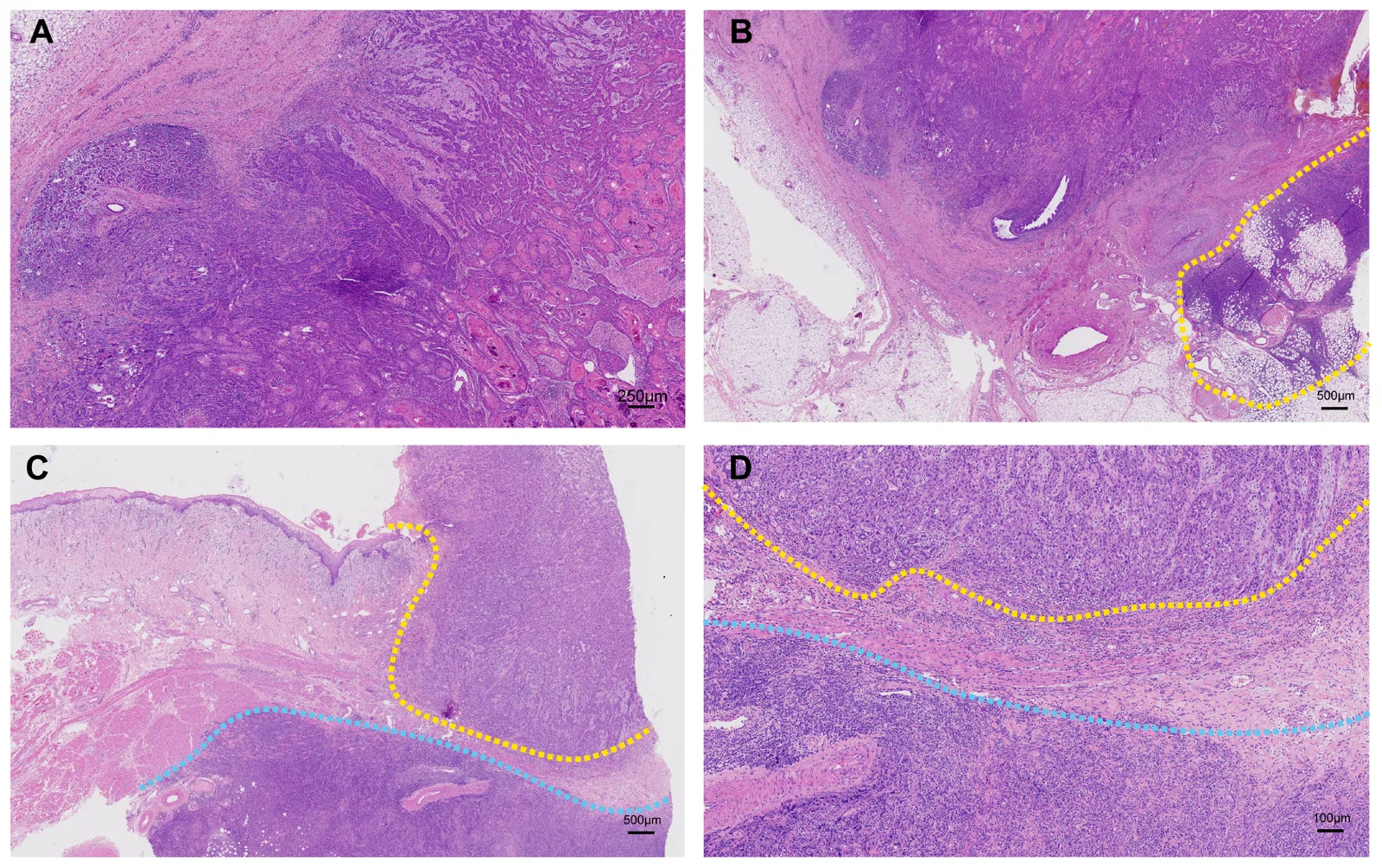
Postoperative histopathological sections of the left buccal lesion. **(A)** Squamous cell carcinoma component: well-to-moderately differentiated squamous cell carcinoma with abundant keratin pearl formation (H&E, 40×). **(B)** The yellow dotted line depicts a diffuse lymphoid cell area that can be seen outside the intrinsic muscle layer of squamous cell carcinoma (H&E, 20×). **(C, D)** Showed more images of squamous cell carcinoma and lymphoma coexisting next to each other after obtained additional specimens. The yellow dashed line depicts the squamous cell carcinoma component above the tissue, and the blue dashed line depicts the lymphoma component below the tissue, with a clear boundary between the two (C H&E, 20×, D H&E, 100×).

**Figure 8:**
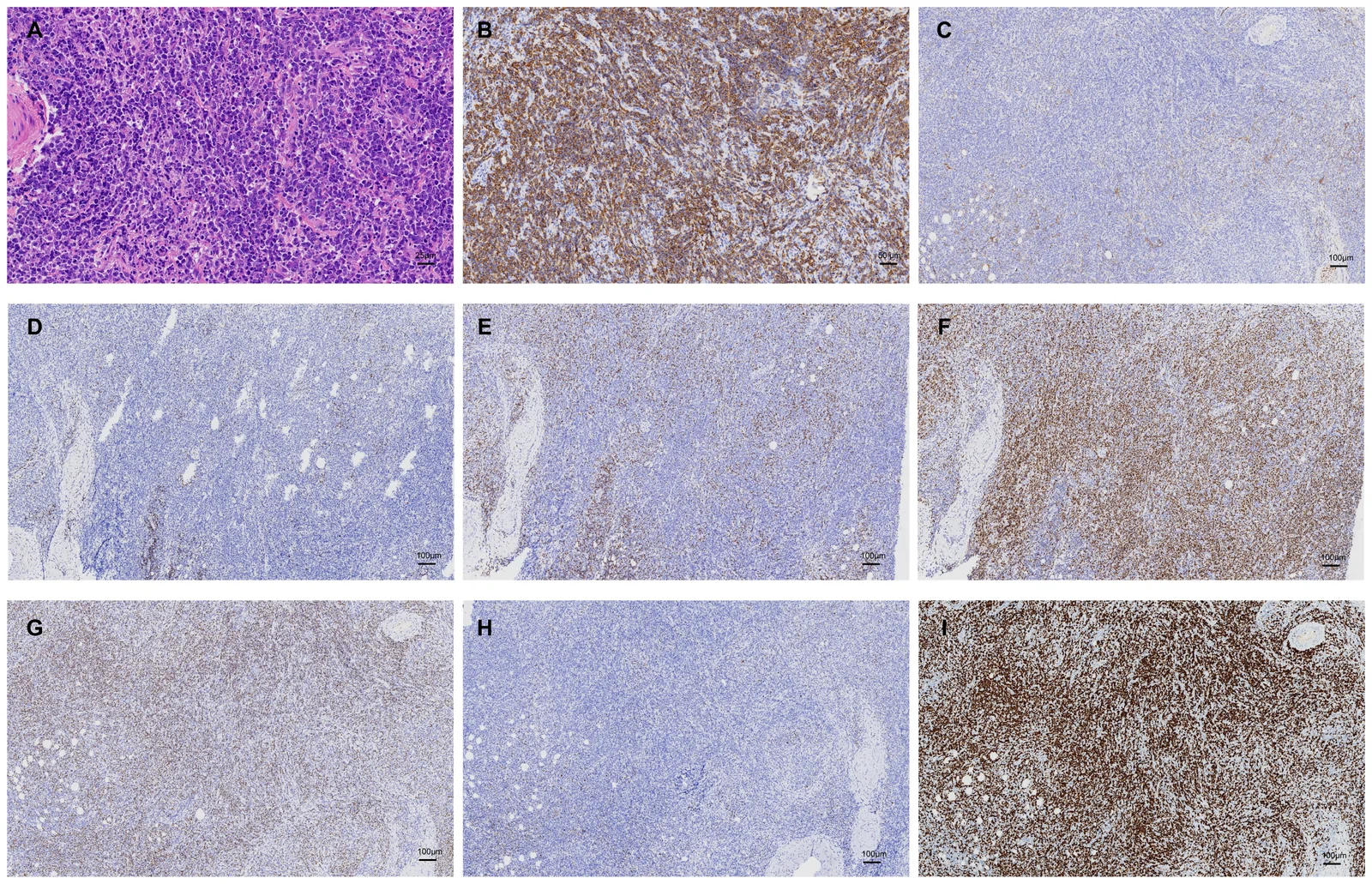
A large panel of antibodies was performed. **(A)** Lymphoma component: Diffuse and patchy sheet-like infiltration of medium-sized to large neoplastic lymphocytes, with poor intercellular adhesion, active and abundant nuclear division (H&E, 400×). **(B)** The lymphoma cells membrane showed strong positivity for CD20 (H&E, 200×). The tumor cells showed negative for CD10 **(C)**BCL-2 **(D)** (H&E, 100×). **(E)** T lymphocytes showd positivity for CD3 (H&E, 100×). The tumor cells showed positivity for BCL-6 **(F)** 、c-Myc **(G)** (H&E, 100×). **(H)** The tumor cells showed scattered positive expression of MUM-1 (H&E, 100×). **(I)** The Ki67 proliferation index was about 70%, showed a high proliferation rate of lymphoma cells (H&E, 100×).

On December 15, 2025, the patient underwent radical resection for left buccal carcinoma combined with cervical lymph node dissection. Intraoperative findings revealed that the left buccal mass invaded the buccinator muscle and extended close to the periosteum. Initial postoperative pathological examination confirmed the primary buccal lesion as typical moderately to well-differentiated SCC with keratin pearl formation (**Figure 7A**). Tumor invasion into the buccinator muscle was noted, alongside focal perineural and lymphovascular invasion. In one slide, however, a focal area of diffuse and relatively uniform atypical lymphoid-cell proliferation was identified in the intrinsic muscle layer deep to the main buccal SCC lesion, raising strong suspicion of a concomitant hematolymphoid tumor (**Figure 7B**). Since the suspected second lesion was deep, obscure, and poorly demarcated, the pathologist’s initial sampling had focused mainly on the buccal SCC and had not adequately sampled the second lesion, making further pathological diagnosis difficult. Following thorough communication between surgical and pathology teams, additional sampling of the surgical specimen was performed. On careful gross examination, the main SCC lesion appeared gray-white, firm, and clearly demarcated from surrounding normal tissue. Deep beneath this tumor, within the intrinsic muscle and adjacent fibroadipose tissue, there appeared to be a paler gray-white, slightly softer area that was poorly demarcated from normal tissue and whose extent could not be accurately assessed grossly. This area was extensively sampled for histologic examination and additional immunohistochemistry (**Figures 7C, D**). The tumor cells in this region were morphologically consistent with diffusely distributed large B lymphocytes, with large irregular nuclei, open chromatin, prominent nucleoli, and frequent mitoses (**Figure 8A**).

Immunohistochemistry (IHC) of the tumor cells showed the following profile: Bcl-2 (–), Bcl-6(60%+), CD10(-), CD19(+), CD20(+), CD21(-), CD3(T cells+), CD30(-), CD5(T cells+), CD7(-), CD79a(+), c-Myc (approximately 50%+), Cyclin D1(-), Ki-67 (approximately 70%), MUM-1(scattered+), p53 (approximately 15%+), and Pax-5(+) (**Figures 8B–I**). *In situ* hybridization for EBER was negative. According to the Hans algorithm, the CD10(-)/Bcl-6(+)/MUM-1(+) profile supported a diagnosis of DLBCL, non-germinal center B-cell-like (non-GCB) subtype, which is associated with a relatively poorer prognosis (19, 20). The two lesions were closely adjacent in space but remained independent and non-fused, consistent with the morpho logic features of a collision tumor (**Figures 7C, D**).

**Figure 9:**
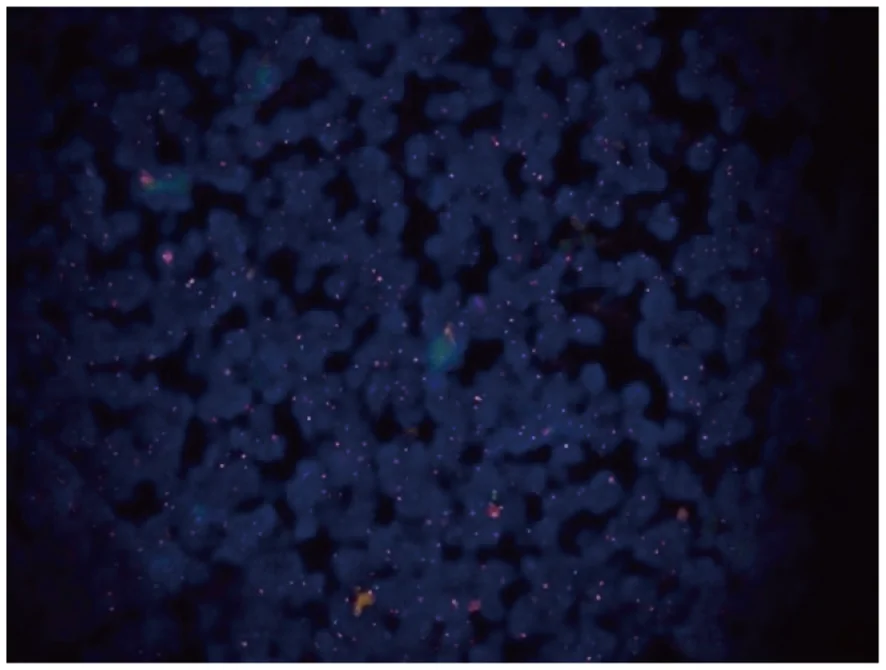
MYC break-apart FISH assay of the buccal neoplasm. DAPI counterstaining marks cell nuclei in blue. Multiple tumor cell nuclei display dissociated red and green fluorescent signals.

Subsequent fluorescence *in situ* hybridization (FISH) demonstrated MYC break-apart rearrangement (**Figure 9**). The proportion of abnormal signals exceeded the laboratory cutoff (1R1G1F, 25%; 1R1F, 8%; 1G1F, 17%). No rearrangements of BCL2 or BCL6 were identified. Molecularly, the case was classified as DLBCL with isolated MYC rearrangement (single-hit DLBCL). Although it did not meet the criteria for high-grade double-hit or triple-hit lymphoma, which carries a particularly poor prognosis, isolated MYC rearrangement remains an independent adverse prognostic factor and often indicates aggressive disease with potential primary resistance to conventional R-CHOP therapy (21, 22). This high-risk molecular feature provided an important biological rationale for subsequent intensified immunochemotherapy with Pola-R-CHP (23).

**Figure 10:**
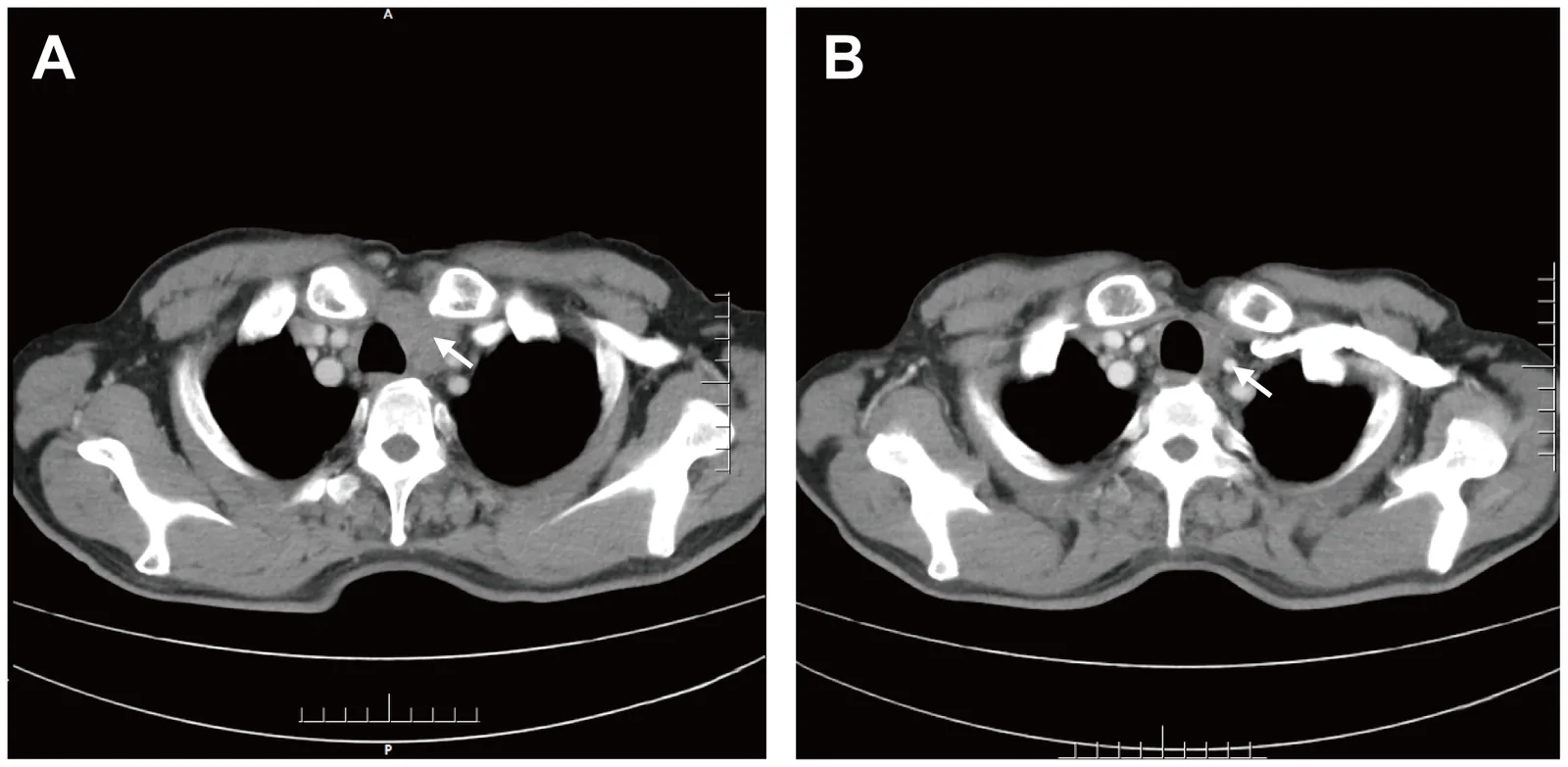
Contrast-enhanced CT of the upper mediastinum at baseline and post-treatment. **(A)** Baseline; **(B)** Two cycles of chemotherapy at the corresponding slice.

After the final postoperative pathological diagnosis, the patient was transferred to the hematology department for further evaluation and systemic treatment planning. Based on the pathological diagnosis of DLBCL and the previous PET-CT findings showing involvement of the buccal soft tissue, bilateral cervical and mediastinal lymph nodes, abdominal lymph nodes, and suspected intestinal lesions, the lymphoma was clinically staged as Ann Arbor stage IV disease. Considering the high-risk non-GCB subtype and MYC-rearranged DLBCL, along with the patient’s advanced age and recent extensive radical surgery for oral cancer, Pola-R-CHP chemotherapy (polatuzumab vedotin, rituximab, cyclophosphamide, doxorubicin, and dexamethasone) was initiated in January 2026. Because PET-CT suggested involvement of the terminal ileum and ascending colon, the clinical team adopted pre-emptive split dosing of cyclophosphamide and doxorubicin to reduce the risk of bowel perforation during intensified immunochemotherapy, allowing gradual tumor regression with close monitoring of abdominal signs. After two cycles of chemotherapy, CT and MRI imaging performed at the end of February 2026 showed shrinkage of multiple enlarged lymph nodes in the bilateral supraclavicular regions, upper mediastinum, and abdomen and pelvis. Thickening of the terminal ileum and ascending colon and diffuse thyroid enlargement were also markedly relieved, confirming a favorable lymphoma response to Pola-R-CHP (**Figure 10**). The overall chemotherapy course was uneventful, with no serious complications. The patient remains clinically stable under regular follow-up.

## Discussion

Collision tumors composed of oral squamous cell carcinoma and DLBCL are rare clinically. Oral squamous cell carcinoma (OSCC) commonly spreads to cervical lymph nodes, with cervical lymph node metastasis reported in approximately 40% of OSCC patients, and occult cervical lymph node metastasis reported in approximately 20%–40% of early-stage oral cancer patients (24, 25). By contrast, lymphoma involving the oral cavity is uncommon: non-Hodgkin lymphoma may present extranodally in approximately 40% of cases, but oral involvement accounts for only a small minority, reported at approximately 0.1%–5% or around 2% in different series (26–28). Their diagnostic difficulty lies mainly in the mutual interference of the two tumor components in clinical presentation and imaging findings. In this case, the presence of buccal SCC made the multiple cervical and mediastinal lymph node lesions easy to interpret as metastatic disease, while their widespread distribution and deviation from conventional lymphatic drainage routes were initially less apparent. This atypical spatial distribution is often caused by the clinically dominant, symptomatic, and aggressive tumor masking another, more occult systemic malignancy, thereby leading to early diagnostic bias and misdirection of clinical management (29, 30).

Only a limited number of head and neck SCC–lymphoma collision or synchronous tumors have been documented in the literature (**Table 2**). Previously reported cases have included metastatic SCC coexisting with B-cell lymphoma in cervical lymph nodes, synchronous head and neck SCC with DLBCL, oral SCC with follicular lymphoma, and nasopharyngeal carcinoma colliding with mantle cell lymphoma. However, most reported cases involved cervical lymph nodes, nasopharyngeal tissue, or anatomically separate synchronous tumors rather than two independent malignant components identified within a buccal surgical specimen. Therefore, the present case appears to be unusual because the epithelial and hematolymphoid components were both identified in the buccal region, remained histologically independent without transition, and required repeated pathological sampling to establish the diagnosis.

**Table 2:** Reported head and neck SCC–lymphoma collision tumors and comparison with the present case.

Study	Presentation	Epithelial tumor	Lymphoid tumor	Relationship between tumors	Key diagnostic issue
Watanabe et al., 2007 (31)	Oropharyngeal lesion with nasopharyngeal lymphoma	Oropharyngeal SCC	Nasopharyngeal malignant lymphoma	Synchronous double malignancy	Cervical lymphadenopathy and head-neck symptoms required distinction between SCC metastasis and lymphoma.
Kakarala et al., 2010 (32)	Cervical lymph node	Metastatic SCC	Low-grade B-cell lymphoma	Collision tumor within cervical lymph node	FNA suggested SCC, but final neck dissection specimen showed both SCC and lymphoma in the same lymph node.
Raldow et al., 2016 (33)	Parotid	Recurrent cutaneous SCC metastatic to parotid	DLBCL	Synchronous SCC and DLBCL	Required integrated multidisciplinary treatment within a short interval; superficial parotidectomy followed by systemic lymphoma therapy.
Jiang et al., 2023 (34)	Nasopharynx	Nasopharyngeal carcinoma	Primary mantle cell lymphoma	True collision tumor in the nasopharynx	Diagnosis depended on pathology plus MRI/PET-CT; authors emphasized that biopsy may miss one component if sampling is incomplete.
Binmadi et al., 2024 (35)	Oral cavity and cervical lymph nodes	Right mandibular OSCC	Nodal follicular lymphoma	Synchronous oral SCC and lymphoma	Lymphoma was detected as a second malignancy in cervical nodes during management of OSCC.
Present case	Buccal region with systemic lesions	Buccal SCC	DLBCL, non-GCB subtype, isolated MYC rearrangement	True collision tumor in buccal surgical specimen	Atypical systemic imaging pattern, Hashimoto’s thyroiditis, and inconclusive small biopsies delayed diagnosis; final diagnosis required extensive postoperative sampling, IHC, and FISH.

Fine-needle aspiration is valuable in the diagnosis of head and neck tumors, but its limitations are evident in the setting of collision tumors or lymphoma (36, 37). In this case, CNB and excision of the thyroid lesion still failed to establish a definitive pathological diagnosis, a difficulty partly attributable to coexisting Hashimoto’s thyroiditis. Several factors may explain the diagnostic challenge of lymphoma in the background of Hashimoto’s thyroiditis. First, the clinical manifestations overlap. Hashimoto’s thyroiditis commonly presents with diffuse, firm thyroid enlargement and may be accompanied by cervical lymphadenopathy, whereas thyroid lymphoma, particularly DLBCL, may also manifest as rapidly progressive, painless thyroid enlargement over a short period. The high similarity in clinical symptoms makes it difficult to distinguish the two solely based on clinical presentation. Second, imaging features overlap. On ultrasound, both Hashimoto’s thyroiditis and thyroid lymphoma may show reduced and heterogeneous thyroid echogenicity, sometimes with posterior acoustic enhancement. Hashimoto’s thyroiditis may produce a reticular or cord-like hyperechoic pattern, and thyroid lymphoma, including MALT lymphoma, may show similar findings, limiting imaging specificity. Third, cytopathologic distinction is difficult. In a thyroid gland affected by Hashimoto’s thyroiditis, FNA has limited accuracy for lymphoma because abundant reactive lymphocytic infiltration makes it difficult to distinguish reactive lymphoid proliferation from neoplastic lymphoid cells. Even CNB, as in this case, may be inconclusive when sampling is insufficient or the disease is unevenly distributed, especially when lymphoma cells are mixed with lymphocytes from Hashimoto’s thyroiditis. The abundant reactive lymphocytes can mask early clonal lymphoma proliferation at the cytologic level, leading to false-negative or indeterminate results (38, 39). Fourth, complexity of immunohistochemical markers. Immunohistochemical markers may present cross-reactivity and overlap between Hashimoto’s thyroiditis and thyroid lymphoma. For example, reactive lymphocytes in some patients with Hashimoto’s thyroiditis may express certain B-cell markers, and lymphoma cells can exhibit similar expression patterns (40). This requires comprehensive interpretation combining multiple markers and clinical context, further increasing diagnostic difficulty. It is important to clarify that the thyroid lesion itself was not finally diagnosed as malignant lymphoma. Although the thyroid specimen showed Hashimoto’s thyroiditis with active lymphoid proliferation and focal atypia, the immunophenotype was not diagnostic of DLBCL and BCR gene rearrangement was negative. Therefore, the thyroid findings were interpreted as autoimmune thyroiditis with reactive and partially atypical lymphoid proliferation, rather than proven thyroid involvement by high-grade lymphoma. The diagnosis of DLBCL in this case was ultimately established from the postoperative buccal surgical specimen, where a separate deep lymphoid tumor component showed the morphology, immunophenotype, and MYC rearrangement consistent with DLBCL. This distinction explains why high-grade lymphoma was not definitively identified in the thyroidectomy specimen and highlights the importance of sampling the most representative lesion when lymphoma is suspected.

Beyond its diagnostic impact, Hashimoto’s thyroiditis may also have biological relevance in this case. Hashimoto’s thyroiditis is characterized by chronic autoimmune inflammation, persistent lymphocytic infiltration, germinal center formation, and a cytokine-rich immune microenvironment within the thyroid gland (41). Such long-standing antigenic stimulation may promote sustained B-cell activation and proliferation and has been implicated in the development of primary thyroid lymphoma, particularly MALT lymphoma and DLBCL (42). In the present patient, the thyroid specimen showed Hashimoto’s thyroiditis with active lymphoid proliferation and atypical lymphoid cells, although B-cell receptor gene rearrangement was negative and thyroid lymphoma could not be confirmed. Therefore, Hashimoto’s thyroiditis should not be interpreted as the direct cause of the buccal DLBCL component. Rather, it may have created a reactive lymphoid microenvironment that mimicked or masked lymphoma, contributed to abnormal thyroid FDG uptake and cervical lymphadenopathy, and complicated the selection and interpretation of biopsy sites.

The amount of tissue obtained by minimally invasive biopsy was limited, and imaging strongly suggested thyroid abnormality. In addition, the surgical team initially had limited awareness of the pathological pitfalls in diagnosing lymphoma. As a result, an ipsilateral thyroid lesion excision was chosen to clarify the nature of the anterior neck mass, rather than direct excisional biopsy of an enlarged cervical lymph node. In retrospect, a targeted CNB or complete excisional biopsy of the most suspicious cervical lymph node would probably have been more appropriate for suspected lymphoma, because nodal architecture and adequate tissue are critical for distinguishing reactive lymphoid proliferation from malignant lymphoma and for performing complete immunohistochemical and molecular assessment. This decision may have delayed a timely definitive diagnosis. This experience suggests that when imaging strongly suggests systemic lymphoma but cytologic or histologic results remain equivocal, surgeons should actively communicate with pathologists and consider the problem from both clinical and pathological perspectives to obtain more diagnostically useful tissue and reduce the risk of a missed diagnosis (43).

Comprehensive, systematic imaging played a central role in the diagnosis of this collision tumor and in the assessment of systemic disease. Ultrasound, CT, and MRI imaging showed early involvement of the thyroid, lower neck, and upper mediastinal lymph nodes in a cross-regional, multicentric distribution. In retrospect, the bilateral cervical lymphadenopathy was an important warning sign, because a unilateral, laterally placed buccal mucosal carcinoma would not be expected to present with prominent contralateral cervical disease as the initial pattern of spread. This pattern was inconsistent with the usual metastatic behavior of advanced buccal SCC and provided important morphologic evidence suggesting a second primary malignancy. Given the unusual early imaging pattern, whole-body 18F-FDG PET-CT should have been considered earlier, once the atypical bilateral cervical, thyroid, and mediastinal distribution was recognized. Earlier PET-CT may have helped define the systemic disease pattern, identify the most metabolically active and diagnostically informative biopsy target, and avoid repeated inconclusive sampling from less representative sites. In this case, 18F-FDG PET-CT revealed discontinuous, multi-organ hypermetabolic lesions and was particularly valuable for defining disease extent and completing tumor staging (44, 45). However, the high sensitivity of imaging also created diagnostic interference: Hashimoto’s thyroiditis may also appear as a markedly hypermetabolic lesion on PET-CT, resulting in false-positive interpretation (46). Such organ-specific reactive changes can obscure the true distribution of disease and influence the choice of initial biopsy site. Looking back, when imaging and repeated biopsies failed to clarify the nature of the anterior neck and intestinal lesions, the clinical team prioritized radical resection of the buccal primary tumor because of extensive local invasion and the risk of potentially fatal bleeding or airway obstruction. Postoperative pathology revealed that the lymphoma was hidden deep within the intrinsic muscle layer and extramuscular fibroadipose tissue of the resected specimen. This diagnostic course suggests that when minimally invasive biopsy is inconclusive and the local lesion carries high-risk features, time-sensitive surgical intervention based on clinical priority can break the diagnostic deadlock. It also underscores the need to move beyond the reflex assumption of single-tumor metastasis and to maintain awareness of rare collision tumors in unusual anatomic settings.

Management of collision tumors must simultaneously account for the marked biological heterogeneity of their distinct tumor components. In this case, the DLBCL component was classified by the Hans algorithm as non-GCB (CD10-/Bcl-6+/MUM-1+) and was confirmed by FISH to harbor an isolated MYC rearrangement. Previous studies have shown that MYC rearrangement is closely associated with aggressive behavior and poor prognosis in DLBCL. Such patients have higher rates of resistance to conventional R-CHOP and limited long-term survival benefit. Based on the therapeutic advance demonstrated in the POLARIX study (47), and after considering the patient’s general condition, advanced-stage DLBCL, non-GCB phenotype, MYC rearrangement, and recent extensive oral cancer surgery, the hematology team selected Pola-R-CHP as the systemic treatment.

Polatuzumab vedotin is an antibody-drug conjugate targeting CD79b and delivers the cytotoxic payload monomethyl auristatin E directly to B-cell lymphoma cells. In the Pola-R-CHP regimen, polatuzumab vedotin replaces the highly neurotoxic vincristine from the conventional R-CHOP regimen. This modification introduces potent targeted tumoricidal activity while avoiding the risk of additive neurotoxicity that may arise from the combination of two microtubule inhibitors (23, 48). This distinction was particularly important in this patient, who had recently undergone extensive maxillofacial tumor resection and delicate flap reconstruction. Avoiding vincristine-associated peripheral neuropathy may help protect flap perfusion and metabolism, facilitate early recovery of mouth opening and swallowing, and reduce the risk of chemotherapy-related paralytic ileus. In addition, the use of liposomal doxorubicin may preserve antitumor efficacy while reducing potential cardiotoxicity in an older patient (49). These decisions illustrate the importance of integrating evidence-based medicine with individualized clinical context in the management of collision tumors. However, the sequential use of surgery and Pola-R-CHP in this patient reflected individualized management rather than evidence for a standardized treatment strategy. Longer follow-up is required before any conclusion can be drawn regarding treatment efficacy, durability of response, or long-term outcome.

## Conclusion

Collision tumors consisting of buccal SCC and DLBCL are rare, clinically complex, and easily mistaken for metastatic progression of a single malignancy. When patients with head and neck SCC present with lesions at multiple sites that do not follow the expected lymphatic drainage pattern, clinicians should maintain a high suspicion for a second primary malignancy. Inflammatory diseases such as Hashimoto’s thyroiditis can show abnormal hypermetabolism on imaging and may confound lesion characterization. In addition, minimally invasive needle biopsy may be limited by sampling constraints and background inflammatory interference when diagnosing hematolymphoid tumors arising in chronically inflamed tissue. When imaging and minimally invasive pathology fail to establish a diagnosis and the local lesion carries a risk of progression, prioritizing surgical control of the primary local tumor can provide an intact specimen for definitive diagnosis. PET-CT, adequate pathological sampling, and multidisciplinary collaboration are essential for accurate diagnosis and rational treatment. The sequential use of surgery and Pola-R-CHP in this patient reflected individualized management rather than evidence for a standardized treatment strategy. Longer follow-up is required before any conclusion can be drawn regarding treatment efficacy, durability of response, or long-term outcome.

## Data Availability

The original contributions presented in the study are included in the article/supplementary material. Further inquiries can be directed to the corresponding author.
